# Hemispheric lateralization of semantic processing before and after aripiprazole treatment in first-episode psychosis or ultra-high risk state

**DOI:** 10.1038/s41537-022-00304-1

**Published:** 2022-12-03

**Authors:** Tzung-Jeng Hwang, Chia-Ta Chan, Cheng-Yu Hsieh, Chih-Min Liu, Chen-Chung Liu, Yi-Ling Chien, Ming H. Hsieh, Yi-Ting Lin, Tai-Li Chou

**Affiliations:** 1 Department of Psychiatry, National Taiwan University Hospital and College of Medicine, Taipei, Taiwan; 2 Neurobiology and Cognitive Science Center, National Taiwan University, Taipei, Taiwan; 3 Shin Kong Wu Ho-Su Memorial Hospital, Taipei, Taiwan; 4 Department of Psychology, National Taiwan University, Taipei, Taiwan; 5 Department of Psychology, Royal Holloway, University of London, Egham, UK

**Keywords:** Psychosis, Psychosis

## Abstract

Whether aberrant language-related lateralization can be improved after antipsychotic treatment in drug-free patients with first-episode psychosis or ultra-high risk state is little known. We aimed to investigate the improvement in lateralization of semantic processing after antipsychotic treatment and associated clinical and cognitive changes. Twenty-one drug-free patients with first-episode psychosis or ultra-high risk state underwent functional magnetic resonance imaging with a semantic task, neuropsychological testing, and clinical assessments with the Positive and Negative Syndrome Scale before and after 6 weeks of aripiprazole treatment. A lateralization index of the region of interest, i.e., inferior frontal gyrus, was calculated and correlated with the behavioral indices of the semantic task, Positive and Negative Syndrome Scale scores, and language-related neuropsychological test scores. After treatment, the lateralization index of the inferior frontal gyrus was significantly increased, which was related to reduced activation of the right inferior frontal gyrus. The increase in the lateralization index was significantly associated with the increase in verbal fluency score. A higher baseline accuracy of the semantic task was associated with a higher post-treatment lateralization index of the inferior frontal gyrus and greater improvement of the total score and positive subscore of the Positive and Negative Syndrome Scale. Our findings indicated aripiprazole treatment significantly increased semantic processing-related lateralization in the inferior frontal gyrus in drug-free patients with first-episode psychosis or ultra-high risk state. A higher baseline accuracy might predict a higher post-treatment lateralization index and greater symptom improvement.

## Introduction

Language disorder is a core symptom of schizophrenia^1^. It impairs a patient’s communicative capacity and can lead to social isolation^2,3^. Crow hypothesized that schizophrenia might result from anomalous language functions related to the failure of hemispheric dominance for language^4^. Language lateralization, or the extent to which the brain is asymmetrical in language-related areas, has been associated with hemispheric dominance. Many anatomical studies using post-mortem autopsy, brain CT, or brain MRI have revealed decreased asymmetry, and, thus lower indices of language lateralization in patients with schizophrenia^4–6^. Functional studies using dichotic listening tests^7^, event-related potential tests^8,9^, and positron emission tomography (PET)^10^, have corroborated this disturbed language-related lateralization. It has been shown that, like PET^11^ and the Wada test^12^, functional MRI with BOLD (blood oxygenation level-dependent) signals can determine hemispheric language dominance with reliability and validity. A few fMRI studies have examined abnormalities of language pathways in patients with schizophrenia, and the most consistent finding has been disturbance of left hemispheric dominance, irrespective of language task^13^.

Reduced language-related lateralization has been identified in fMRI in both male^14^ and female^15^ schizophrenia patients. It has also been observed irrespective of illness duration in both chronic patients^14,16–19^ and first-episode patients^20,21^. More specifically, some studies have reported reduced language-related lateralization in first-episode patients regardless of whether the patients were treatment-naïve or undergoing initial antipsychotic treatment^20,21^. Moreover, Razafimandimby et al. reported stable reduced language-related lateralization for over 2 years in chronic patients^22^. However, no prior study has investigated whether disturbed language-related lateralization occurs in subjects with first-episode psychosis (FEP) or ultra-high risk state (UHR) and whether such disturbance can be improved after antipsychotic treatment. In addition, although previous studies have used various language cognitive tests to study improvements in the language function of patients with schizophrenia after antipsychotic treatment^23–25^, no studies have combined fMRI to do so in antipsychotic-free patients.

This study aimed to investigate the improvement of language-related lateralization after 6 weeks of aripiprazole treatment by using fMRI in antipsychotic-free subjects with FEP or UHR. A semantic judgment task with concurrent fMRI has been proposed to be an ideal tool to assess language-related lateralization^26^. Previous fMRI studies using this task have reported greater activation in semantic networks, particularly in the left inferior frontal gyrus (IFG, BA45) in normal adults^27,28^, normal children^29^, and patients with schizophrenia^30,31^. An age-related increase in left IFG activation has consistently been reported during semantic processing^32–34^. In contrast, a deficit in semantic processing has been associated with greater activation in the right IFG in patients with schizophrenia^35^. We thus chose IFG as the region of interest and hypothesized that, before treatment with aripiprazole, antipsychotic-free subjects with FEP or UHR may exhibit greater activation in the right IFG, and that after treatment, the aberrant language-related lateralization may be improved, as reflected by an increase in lateralization index (LI). We also hypothesized that these treatment-related fMRI changes might be correlated with improvements in behavioral performance in the semantic judgment task, clinical symptoms, and language-related neuropsychological measures.

## Results

### Subjects

Twenty-one antipsychotic-free patients (FEP *n* = 15, and UHR *n* = 6) and 21 normal controls were enrolled in this study. All patients had varying degrees of psychotic symptoms. Among the patients, nine were drug-naïve and 12 had a short exposure to antipsychotics. The mean average interval between the two fMRI tests was 6 weeks in both the patient and control groups. The final average dosage of aripiprazole was 7.25 mg/day. After 2 to 5 years (mean: 2.9 years) of follow-up, 16 patients were diagnosed as having schizophrenia, three with schizophreniform disorder, one with delusional disorder, and one with UHR (because of loss of follow-up), indicating 95% (20/21) of this sample belonged to the early stage of the schizophrenia-spectrum disorder.

The demographic and clinical characteristics are shown in Table 1. There were significant differences in years of education and IQ between the patient and control groups (*p* < 0.01). There were significant improvements in the PANSS-total score, three subscores, and the dimension score of delusion/hallucination after aripiprazole treatment in the patient group (all *p* values <0.001), but not in verbal IQ or verbal fluency score (Table 2).

**Table 1:** Comparisons of demographic characteristics between the patients (FEP or UHR) and controls.

	Patients (*n* = 21)	Controls (*n* = 21)	*p* value^a^
Gender (M/F)	11/10	11/10	1.000
Age (years), mean (SD)	27.6 (7.4)	27.3 (4.3)	0.880
Education (years), mean (SD)	14.0 (2.6)	16.0 (1.4)	0.003
Handedness (L/R)^c^	1/20	0/21	0.311
Full IQ, mean (SD)	92.7 (16.9)^b^	112.7 (8.8)	<0.001

*FEP* first-episode psychosis, *UHR* ultra-high risk state, *SD* standard deviation.

^a^Comparison between patients and controls with chi-square test or two-sample *t*-test.

^b^Post-treatment Full IQ for only 20 patients.

^c^Measured with the Edinburgh Handedness Inventory.

**Table 2:** Comparisons of clinical and neuropsychological measurements in patients with FEP or UHR before and after aripiprazole treatment.

	Pretreatment (*n* = 21) Mean (SD)	Post-treatment (*n* = 21) Mean (SD)	*t* value^a^	*p* value^a^
PANSS-total score	71.8 (16.3)	46.5 (10.5)	*t*(20) = −7.81	<0.001
PANSS-positive score	19.8 (4.8)	10.9 (2.8)	*t*(20) = −7.02	<0.001
PANSS-negative score	14.8 (6.1)	11.1 (3.3)	*t*(20) = −4.84	<0.001
PANSS-general psychopathology score	37.2 (8.5)	24.4 (7.3)	*t*(20) = −7.94	<0.001
Delusion/hallucination dimension score^b^	16.0 (3.9)	8.1 (3.1)	*t*(20) = −8.39	<0.001
Verbal IQ^c^	36.0 (12.4)	37.6 (12.5)	*t*(20) = 0.27	0.792
Categorical verbal fluency score	31.9 (7.9)	32.2 (6.4)	*t*(20) = 1.57	0.132

*FEP* first-episode psychosis, *UHR* ultra-high risk state, *SD* standard deviation, *PANSS* positive and negative syndrome Scale.

^a^Comparing the difference between post- versus pretreatment using paired *t*-test.

^b^Composed of “Hallucinatory behaviors” (P3), “Unusual thought content” (G9), “Delusions” (P1), and “Suspiciousness/persecution” (P6).

^c^Sum of scale scores of four verbal IQ items (information, arithmetic, similarity, digit span).

### Behavioral results

Regarding the semantic judgment task, there was no significant post- versus pretreatment change in reaction times in the patient group (*t*(20) = 0.37, *p* = 0.714) (Table 3). There was a significant post- versus pretreatment improvement in accuracy in the patient group (*t*(20) = 2.15, *p* = 0.044) (Table 3).

**Table 3:** Comparisons of behavioral performances on the semantic judgment task in patients with FEP or UHR before and after aripiprazole treatment.

		Pretreatment patients (*n* = 21) Mean (SD)	Post-treatment patients (*n* = 21) Mean (SD)
Accuracy (%)^a^	Related	79.3 (20.4)	87.0 (12.4)
	Unrelated	89.5 (16.6)	96.0 (6.7)
	Simple	95.5 (12.6)	98.0 (4.3)
	Control	92.9 (16.6)	97.6 (5.7)
Reaction times (ms)	Related	1033 (175)	1052 (188)
	Unrelated	1027 (191)	1011 (192)
	Simple	721 (110)	738 (137)
	Control	704 (143)	714 (167)

*FEP* first-episode psychosis, *UHR* ultra-high risk state, *SD* standard deviation.

^a^*p* = 0.044, *t*(20) = 2.15, post- versus pretreatment.

### Imaging results

**Fig. 1:**
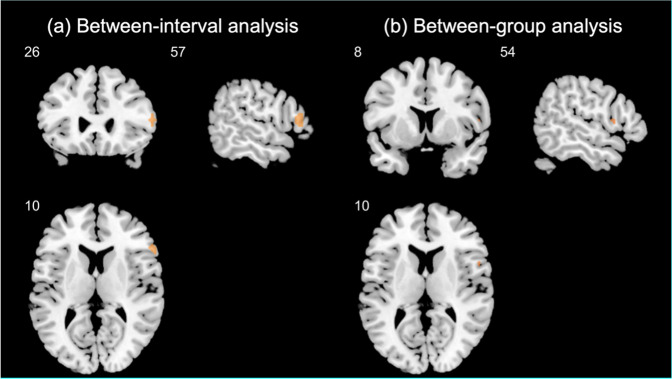
Between-interval and between-group analysis. **a** Between-interval ROI analysis was conducted in the patients before and after aripiprazole treatment, with adjustment of age, sex, education, state (FEP/UHR), and aripiprazole dosage. **b** Between-group ROI analysis was conducted between the patients and normal controls at baseline, with adjustment of education. The right IFG activation during the semantic task (related condition vs. perceptual control) was significant using *p* < 0.05 FWE (family-wise error) corrected with the use of an anatomical mask of the right IFG from the WFU PickAtlas toolbox. ROI region of interest, IFG inferior frontal gyrus, FEP first-episode psychosis, UHR ultra-high risk state.

Table 4 presents a direct comparison of the pre- versus post-treatment in the patient group at a whole-brain analysis. Furthermore, the ROI analysis showed significant activation in the right IFG (BA45, peak MNI coordinates = [60, 23, 10], cluster size = 1, *z* value = 3.48, *p* < 0.05 FWE-corrected). Additional ROI analysis in which age, sex, education, status (FEP/UHR), and aripiprazole dosage were controlled also showed significant activation in the right IFG (BA45, peak MNI coordinates = [57, 26, 10], cluster size = 18, *z* value = 3.80, *p* < 0.05 FWE-corrected; Fig. 1a). This finding revealed that the reduction in right IFG after treatment was still robust after adjusting for related demographic and clinical variables.

**Table 4:** Between-interval whole-brain analysis (pretreatment vs. post-treatment) for patients and between-group whole-brain analysis (patients vs. normal control at baseline) of brain activation during the semantic task (related condition vs. perceptual control).

Cortical Regions	H	BA	Voxels	z-test	MNI Coordinates		
					*x*	*y*	*z*
**Between-interval analysis for patients with FEP or UHR (*n*** = **21)**							
*pretreatment* > *post-treatment*							
Caudate	R	NA	11587	4.92	6	14	4
Caudate	L	NA		4.72	−18	14	−8
Superior Temporal Gyrus	R	22		4.28	48	−13	−5
Inferior Frontal Gyrus	R	44/45	26	3.48	60	23	10
Superior Frontal Gyrus	R	6	66	3.18	12	−1	61
Superior Temporal Gyrus	R	22	33	2.82	−6	−76	19
*post-treatment* > *pretreatment*							
None							
**Between-group analysis (*n*** = **21 for each group) at baseline** *patients with FEP or UHR at pretreatment* > *normal controls*							
Insula	R	31	149	3.60	36	17	4
Inferior Frontal Gyrus	R	44/45		3.12	54	8	10
Postcentral Gyrus	L	2	35	3.55	−48	−31	43
Middle Frontal Gyrus	R	10	30	3.14	42	44	25
Precuneus	R	7	69	3.09	24	−70	22
Superior Frontal Gyrus	R	10	35	3.08	30	50	19
Caudate	R	NA	30	3.00	9	11	−2
Insula	L	13	15	2.92	−39	−10	13

*FEP* first-episode psychosis, *UHR* ultra-high risk state, *H* hemisphere, *L* left, *R* right, *BA* Brodmann’s area. Coordinates of activation peak(s) within a region based on a *z*-test are given in the MNI stereotactic space (x, y, z); Voxels: number of voxels in a cluster at *p* < 0.005 (uncorrected) with a cluster size greater than or equal to 10 voxels at a whole-brain analysis.

In addition, Table 4 presents a comparison between the patient and control group with whole-brain analysis at baseline. In particular, the ROI analysis showed significant activation in the right IFG (BA45, peak MNI coordinates = [54, 8, 10], cluster size = 2, *z* value = 3.12, *p* < 0.05 FWE-corrected). To avoid potential confounding from significant group differences in education and full-scale IQ, we conducted an additional ROI analysis with an adjustment of education due to a high correlation between the two variables (*r* = 0.57, *p* = 0.006). Significant activation in the right IFG was also revealed (BA45, peak MNI coordinates = [54, 8, 10], cluster size = 5, *z* value = 3.17, *p* < 0.05 FWE-corrected; Fig. 1b). In contrast, there was no significant activation in the right IFG for the first versus second-time comparison in the controls (*n* = 6) who received repeated fMRI examinations (Supplementary Table S1).

The pretreatment LI of the IFG was 0.54 in the patient group, and the post-treatment LI increased to 0.72. The paired-sample *t*-test showed a significant difference between the two time points (*t*(20) = −2.36, *p* = 0.029). In addition, the LI value for the control group at the baseline (*n* = 21) was 0.68. No significant group differences were found when we compared the control group with the patient group either at pretreatment (*t*(40) = 0.87, *p* = 0.388), or at post-treatment (*t*(40) = −0.29, *p* = 0.770).

To clarify whether the increase of LI was related to changes in activation in the left IFG, right IFG, or both, the change in LI was correlated with the changes in voxel values for the right or left IFG. A significant correlation was found for the right IFG (Spearman’s rho = −0.67, *p* = 0.001), but not for the left IFG (Spearman’s rho = −0.05, *p* = 0.841), implying that the increase of LI was related to the reduced activation of the right IFG after treatment.

### Correlation results

After excluding potential outliers based on Mahalanobis distance (corresponding *p* value <0.001) and Cook’s distance (<1)^36^, scatter plots for the following correlations are presented in Supplementary Fig. S1. In the patient group, the improvement in the categorical verbal fluency score (pre- versus post-treatment) was positively correlated with the change in LI (pre- versus post-treatment) of the IFG (Spearman’s rho = 0.45, *p* = 0.043)(Fig. S1a). The accuracy of the semantic judgment task at the baseline was positively correlated with the post-treatment LI of the IFG (Spearman’s rho = 0.47, *p* = 0.030) after excluding one outlier (Fig. S1b1), and negatively correlated with the reduction (pre- versus post-treatment) in the PANSS-total score (Spearman’s rho = −0.49, *p* = 0.023), positive subscore (Spearman’s rho = −0.58, *p* = 0.006), and delusion/hallucination dimensional subscore (Spearman’s rho = −0.60, *p* = 0.004)(Fig. S1b2–b4). Furthermore, the change in accuracy of the semantic judgment task (pre- versus post-treatment) was negatively correlated with the reduction (pre- versus post-treatment) in the PANSS-total score (Spearman’s rho = −0.53, *p* = 0.013), positive subscore (Spearman’s rho = −0.50, *p* = 0.022), and general psychopathology subscore (Spearman’s rho = −0.61, *p* = 0.003)(Fig. S1c1–c3).

## Discussion

To the best of our knowledge, this is the first study to use an fMRI examination with a semantic judgment task to investigate the effect of 6-week aripiprazole treatment on language-related lateralization in patients with FEP or UHR. Longitudinal evaluations with the PANSS and language-related NPT further strengthened the study. There are three main findings. First, the aberrant language-related lateralization in the IFG could be increased after treatment with aripiprazole. Second, the post-treatment increase in LI was positively correlated with an improvement in categorical verbal fluency scores. Third, the accuracy of the semantic judgment task at baseline was positively correlated with the post-treatment LI of the IFG and reductions in total score, positive subscore, and delusion/hallucination dimensional score of the PANSS. In addition, the post-treatment increase in accuracy was positively correlated with decreases in the total score, positive subscore, and general psychopathology subscore of the PANSS.

Deficits in language-related pathways are clinically important because a language disorder itself is one of the core symptoms of schizophrenia, and the deficits are related to delusions/hallucinations^13,37^. Decreased language lateralization has been reported in patients with schizophrenia, with no differences in the reduction in leftward lateralization over 2 years in chronically medicated patients^13,22^. Only one study has examined language-related lateralization in drug-naive first-episode patients with schizophrenia, and the results showed reduced lateralization^21^. Another study in first-episode medicated patients also showed decreased language-related lateralization after treatment with antipsychotics for 1 to 4 weeks^20^. Because both studies were cross-sectional, whether the aberrant language lateralization could be improved after antipsychotic treatment was still unknown. The novel finding of our study is that this functional aberrancy of the IFG could be significantly improved after a 6-week aripiprazole treatment in drug-free patients with FEP or UHR.

Many previous studies concerning the language-related pathway in schizophrenia have reported impaired functional and anatomical connectivity of the IFG, the primary region of interest of the current study^13,35,38–41^. A significant association between auditory verbal hallucinations and right middle/inferior frontal activation has also been reported^42,43^. We found that the significant increase in LI after 6 weeks of treatment was correlated with the decrease in right IFG activation and improvement in categorical verbal fluency scores. These correlations suggest that the increase in lateralization was mainly due to enhanced right IFG activation after treatment, and that such changes in lateralization may underpin the improvement in performance on verbal fluency. Previous studies have shown that patients with schizophrenia have impaired verbal fluency, and that this is related to aberrant rightward lateralization in the frontal cortex^44,45^. Abnormal semantic memory (such as impaired verbal fluency) can be improved by atypical antipsychotic treatment (especially those with 5-HT_1A_ agonist activity such as aripiprazole)^23^. Our findings support that the underlying mechanism of this semantic memory improvement after atypical antipsychotic treatment may be through the increase of IFG lateralization.

Many previous studies have shown a significant LI difference between schizophrenia patients (acute or chronic) and normal controls during a language or semantic processing task^10,13–21,44^. The current study showed no significant difference in LI between the patient group (either pre- or post-treatment) and the control group (at baseline). These inconsistencies could be related to several factors, including different disease stages (very early stage of the illness), potential selection bias (for example, those patients with more severe psychotic symptoms might refuse to join), different semantic tasks, or patients’ motivation to perform the task, etc. Nevertheless, as shown in Table 4, there was a significant difference in right IFG activation between the patient and control group at baseline, and within the patient group before and after treatment. This significant decrease in right IFG activation led to a significant LI increase after treatment. The post-treatment change in LI was correlated with the change in verbal fluency score, suggesting LI of IFG might be linked to frontal cognitive function. This finding is compatible with a previous study showing that verbal fluency performance was related to the activation of IFG^46^.

The study showed that baseline accuracy of the semantic judgment task was associated with the post-treatment LI and reduced severity of positive symptoms. The increase in accuracy of the semantic judgment task was also correlated with decreased severity of positive and general psychopathology symptoms. Since the right middle/inferior frontal gyrus activity has been reported to be positively related to the severity of auditory verbal hallucinations^42,43^, our findings imply that excessive right IFG activation can be improved with aripiprazole, and this improvement is coupled with improved severity of positive symptoms after treatment. In addition, the baseline accuracy of the semantic judgment task could be a potential predictor of clinical symptom improvement and post-treatment lateralization in patients with FEP or UHR. If this finding can be replicated in future studies with a larger sample size, then the baseline accuracy can be a predictor for treatment response. It implies that patients with better cognitive function respond better to antipsychotic treatment through the increase of brain lateralization.

Normal controls performed the same semantic task twice in the 6-week study period, and more cognitive resources were recruited the first time, as reflected by the before-versus-after analysis (Supplementary Table S1). These timing-related changes may be due to general priming effects on attention or memory processing. Similar topographic activation in the patient group was also observed, including more activation in the right cingulate and superior temporal gyrus at baseline. But the significant between-interval difference in right IFG activation of the patient group could not be attributed entirely to the repetition effect since no such difference was observed in the control group.

The clinical response to antipsychotic treatment has been related to dopamine D2 receptor occupancy in the bilateral striatum in schizophrenia^47^. In our study, all patients showed a satisfactory response to aripiprazole treatment, suggesting that their striatal dopamine activity might have been inhibited. These findings are compatible with our fMRI results showing decreased bilateral caudate activation after 6 weeks of aripiprazole treatment (Table 4).

There are several limitations to this study. First, the sample size was not large, limiting the statistical power for analyses of language-related function and generalizability. Only 6 controls received two fMRI examinations, so there might be limited power to show the between-interval difference. Second, although the two groups were not significantly different in sex, age, and handedness, the years of education and full IQ scores in the control group were superior to those of the patient group. Thus, they may have recruited fewer brain resources to perform the semantic judgment task during the fMRI scan. However, this effect could be minimized due to the longitudinal design with within-group comparisons. Third, because the sample size is relatively small, caution has to be exercised in interpreting these preliminary correlational results, and more studies with a larger sample size are warranted for verification. Fourth, one UHR patient was lost to follow-up; thus, we did not know the definite diagnosis. Nevertheless, 95% (20/21) of the whole sample belonged to the early stage of the schizophrenia-spectrum disorder.

In conclusion, this is the first study to report a significant improvement of aberrant semantic processing-related lateralization after 6 weeks of aripiprazole treatment in antipsychotic-free patients with FEP or UHR, and this was closely related to reduced activation of the right IFG. The significant increase in IFG lateralization was associated with improved performance in verbal fluency. Moreover, the accuracy of the semantic judgment task at baseline might predict the post-treatment lateralization of the IFG and the reduction in clinical symptom severity. Future studies may examine whether this semantic judgment task can be useful in predicting clinical treatment response in the early phase of the schizophrenia-spectrum disorder.

## Methods

This study was an open-label clinical trial in which subjects with FEP or UHR received a flexible dose of aripiprazole for 6 weeks between 2008 and 2020. The subjects were consecutive patients recruited from the outpatient clinics of the Department of Psychiatry, National Taiwan University Hospital. To join the study, patients had to understand the procedures and agree to sign the informed consent. Related clinical trial procedures have been described in our previous publications^48,49^. The Institutional Review Board approved the study protocol and procedures at National Taiwan University Hospital.

### Subjects and study design

The FEP subjects were those who developed full-blown psychosis and fulfilled the *Diagnostic and Statistical Manual of Mental Disorders, Fourth Edition, Text Revision* (*DSM-IV-TR*)^50^ criteria for schizophrenia, schizophreniform disorder, or delusional disorder within 1 year. The UHR subjects showed subthreshold psychotic symptoms and fulfilled the comprehensive assessment of at-risk mental status criteria either with attenuated psychotic symptoms or with brief limited intermittent psychotic symptoms^51^. “Drug-naïve” was defined as subjects who had never received antipsychotic treatment before, although they may have received anxiolytic, hypnotic, or antidepressant agents. Subjects who received any antipsychotic treatment for less than 3 months were defined as having a short exposure to antipsychotics.

All FEP and UHR subjects met the following inclusion criteria: (1) native Mandarin-Chinese speakers, (2) normal or corrected-to-normal vision, (3) no active substance use problems in the 6 months before the study, (4) no neurological disorders, such as epilepsy, stroke, etc., and (5) no major systematic physical illnesses. The patients who had had a psychotic episode more than 1 year previously were excluded. The subjects with short exposure to antipsychotics were asked not to take any antipsychotics for at least 1 week before baseline assessments. If necessary, ancillary lorazepam (no more than 2 mg/day) or estazolam (no more than 2 mg/day) could be prescribed for supportive treatment. A total of 21 subjects with FEP or UHR were enrolled, all of whom received fMRI examinations with a semantic judgment task, neuropsychological testing (NPT), and clinical assessments with the Chinese version of the Positive and Negative Syndrome Scale (PANSS)^52^ before and after 6 weeks of aripiprazole treatment.

Twenty-one normal controls were also recruited through advertisements, and they received interviews to ensure that they had no current or lifetime major psychiatric, neurological, or medical illnesses. The controls also received the NPT and fMRI semantic task assessments at baseline. Six of them repeated the same fMRI assessment 6 weeks later without receiving any intervention.

### Functional activation task: the semantic judgment task

Two Chinese characters were presented visually in sequence, and the subjects had to determine whether they were related in meaning^28^. The subjects were instructed to quickly and accurately press with their right hand the “yes” button if the cards were related and the “no” button if they were unrelated. In the perceptual control condition, 24 pairs of non-characters were used. The subjects were instructed to determine whether or not the pair of non-characters was identical by pressing the “yes” or “no” button above. The related condition was used to investigate semantic processing^28^, and the perceptual control condition was used to control for non-language components such as response demands at baseline^29^. The stimulus characteristics of the related and unrelated conditions (see supplementary methods) were the same as those in our prior study^28^.

### MRI data acquisition

The subjects lay in the scanner with their head position secured. An optical response box was placed in the subject’s right hand, and the head coil was positioned over the head. The subjects viewed visual stimuli projected onto a screen via a mirror attached to the inside of the head coil. In this study, we used an event-related design. Each subject performed two functional runs, each of which took 4.7 min and had 136 image volumes.

All images were acquired using a 3 Tesla Siemens Trio scanner with a 32-channel head coil. Gradient-echo localizer images were acquired to determine the placement of the functional slices. Functional images were acquired with the echo planar imaging method to detect BOLD (blood oxygenation level-dependent) signals. The scanning parameters were as follows: repetition time (TR) = 2000 ms; echo time (TE) = 24 ms; flip angle = 90°; matrix size = 64 × 64; field of view = 25.6 cm; slice thickness = 3 mm; number of slices = 34. We acquired 272 images. A high-resolution, T1-weighted three-dimensional (3D) image was also acquired (Magnetization Prepared Rapid Gradient Echo, MP-RAGE; TR = 1560 ms; TE = 3.68 ms; flip angle = 15°; matrix size = 256 × 256; field of view = 25.6 cm; slice thickness = 1 mm). The orientation of the 3D image was identical to the functional slices. The task was administered in a pseudorandom order for all subjects, and the order was optimized for an event-related design. We used the Optseq script (https://surfer.nmr.mgh.harvard.edu/optseq/) for a randomized event-related design which implemented the optimal approach^53^. The script has also been used in our prior research on schizophrenia^30,35^.

### Behavioral data analysis

The paired *t*-test was used to analyze behavioral data (i.e., accuracy and reaction times) for performance on the related condition in the semantic judgment task with SPSS version 23 (IBM Corp., Armonk, NY).

### Imaging data analysis

Data analysis was performed using SPM8 (Statistical Parametric Mapping) (Wellcome Institute, London, UK). The functional images were corrected for differences in slice acquisition time to the middle volume and realigned to the first volume in the scanning session using affine transformations. No subject had more than 3 mm of movement in any plane. Co-registered images were normalized to the Montreal Neurological Institute (MNI) average template. Statistical analyses were performed on the smoothed data (6 mm isotropic Gaussian kernel) with a high pass filter (128 s cutoff period) in order to remove low-frequency artifacts.

Data from each subject were entered into a general linear model using an event-related analysis procedure. Character pairs were treated as individual events and modeled with the onset of response latency using a canonical hemodynamic response function (HRF). Parameter estimates from contrasts of the canonical HRF in single-subject models were entered into random-effects analysis using one-sample *t*-tests across all participants to determine whether activation during a contrast was significant (i.e., parameter estimates were reliably greater than 0). For the contrast within each group, we compared the related condition to the perceptual condition to observe semantic processing^30^. For each group, we performed paired *t*-tests for the related versus perceptual contrast to capture the changes within 6 weeks. Reported areas of activation were defined as being significant when *p* < 0.005 uncorrected at the voxel level, with a cluster size ≥10 voxels in a whole-brain analysis. Region of interest (i.e., IFG) analyses were conducted using an anatomical mask from the WFU PickAtlas toolbox (https://www.nitrc.org/projects/wfu_pickatlas/), and the areas of activation were defined as being significant when *p* < 0.05 family-wise error (FWE)-corrected in the IFG. To avoid potential confounding from demographic variables, we also conducted additional ROI analyses in which demographic and clinical characteristics were controlled.

To test our hypothesis that language-related lateralization may be disturbed in patients with FEP or UHR, we computed the LI using the LI-toolbox^54^ on contrast maps of the related versus perceptual conditions for each subject. For each map, the mean intensity of the voxels in the image served as the threshold (i.e., an “adaptive threshold” in LI-toolbox) under the assumption that meaningful responses would be above average intensity. Voxel values exceeding such an internal threshold were summed to compute a global value for bilateral anatomical masks, i.e., the IFG. These values were then used to compute an LI value for each patient, with the formula: LI = (left – right)/(left + right). The LI value ranged between +1 and −1, with +1 indicating pure left lateralization and −1 pure right lateralization.

### Correlation analysis

Spearman’s rank correlations were used to explore relationships among the treatment-related changes, including LI, behavioral results (i.e., accuracy and reaction times), PANSS-total score and three subscores (positive, negative, general psychopathology), and language-related NPT scores. Moreover, we used the score of the “delusion/hallucination” dimension composed of “Hallucinatory behaviors” (P3), “Unusual thought content” (G9), “Delusions” (P1), and “Suspiciousness/persecution” (P6) based on the findings from our previous study^55^. The language-related NPT items included: (1) the summation of scale scores of four items (information, arithmetic, similarity, digit span) in the verbal IQ, and (2) scores of the categorical verbal fluency tests. Since the small sample size was vulnerable to outliers, we excluded potential outliers based on Mahalanobis distance (corresponding *p* value <0.001) and Cook’s distance (<1)^36^.

## Supplementary information

Hemispheric lateralization of semantic processing before and after aripiprazole treatment in first-episode psychosis or ultra-high risk state

## Data Availability

The data that support the findings of this study is available from the corresponding author upon reasonable request. The terms of informed consent dating from 2008 do not permit making the data publicly available.

## References

[1] DeLisi LE (2001). Speech disorder in schizophrenia. Schizophr. Bull..

[2] Green MF, Kern RS, Braff DL, Mintz J (2000). Neurocognitive deficits and functional outcome in schizophrenia: are we measuring the “right stuff”?. Schizophr. Bull..

[3] Green MF, Kern RS, Heaton RK (2004). Longitudinal studies of cognition and functional outcome in schizophrenia: implications for MATRICS. Schizophr. Res..

[4] Crow TJ (1997). Schizophrenia as failure of hemispheric dominance for language. Trends Neurosci..

[5] Oertel-Knochel V, Knochel C, Stablein M, Linden DE (2012). Abnormal functional and structural asymmetry as biomarker for schizophrenia. Curr. Top. Med. Chem..

[6] Sommer I, Ramsey N, Kahn R, Aleman A, Bouma A (2001). Handedness, language lateralisation and anatomical asymmetry in schizophrenia: meta-analysis. Br. J. Psychiatry.

[7] Ocklenburg S (2013). Cholecystokinin A receptor (CCKAR) gene variation is associated with language lateralization. PLoS ONE.

[8] Angrilli A (2009). Schizophrenia as failure of left hemispheric dominance for the phonological component of language. PLoS ONE.

[9] Spironelli C, Angrilli A, Stegagno L (2008). Failure of language lateralization in schizophrenia patients: an ERP study on early linguistic components. J. Psychiatry Neurosci..

[10] Artiges E (2000). Altered hemispheric functional dominance during word generation in negative schizophrenia. Schizophr. Bull.

[11] Xiong J, Rao S, Gao JH, Woldorff M, Fox PT (1998). Evaluation of hemispheric dominance for language using functional MRI: a comparison with positron emission tomography. Hum. Brain Mapp..

[12] Dym RJ, Burns J, Freeman K, Lipton ML (2011). Is functional MR imaging assessment of hemispheric language dominance as good as the Wada test?: a meta-analysis. Radiology.

[13] Li X, Branch CA, DeLisi LE (2009). Language pathway abnormalities in schizophrenia: a review of fMRI and other imaging studies. Curr. Opin. Psychiatry.

[14] Sommer IE, Ramsey NF, Kahn RS (2001). Language lateralization in schizophrenia, an fMRI study. Schizophr. Res..

[15] Sommer IEC, Ramsey NF, Mandl RCW, Kahn RS (2003). Language lateralization in female patients with schizophrenia: an fMRI study. Schizophr. Res..

[16] Bleich-Cohen M (2012). Diminished language lateralization in schizophrenia corresponds to impaired inter-hemispheric functional connectivity. Schizophr. Res..

[17] Dollfus S (2005). Atypical hemispheric specialization for language in right-handed schizophrenia patients. Biol. Psychiatry.

[18] Kircher TT (2002). Reversed lateralization of temporal activation during speech production in thought disordered patients with schizophrenia. Psychol. Med..

[19] Li X (2007). fMRI study of language activation in schizophrenia, schizoaffective disorder and in individuals genetically at high risk. Schizophr. Res..

[20] Bleich-Cohen M, Hendler T, Kotler M, Strous RD (2009). Reduced language lateralization in first-episode schizophrenia: an fMRI index of functional asymmetry. Psychiatry Res..

[21] van Veelen NM (2011). Reduced language lateralization in first-episode medication-naive schizophrenia. Schizophr. Res..

[22] Razafimandimby A (2007). Stability of functional language lateralization over time in schizophrenia patients. Schizophrenia Res..

[23] Sumiyoshi C, Sumiyoshi T, Roy A, Jayathilake K, Meltzer HY (2006). Atypical antipsychotic drugs and organization of long-term semantic memory: multidimensional scaling and cluster analyses of category fluency performance in schizophrenia. Int. J. Neuropsychopharmcol..

[24] Bervoets C (2012). Effect of aripiprazole on verbal memory and fluency in schizophrenic patients. CNS Drugs.

[25] Goldberg TE (2000). Effects of neuroleptic medications on speech disorganization in schizophrenia: biasing associative networks towards meaning. Psychol. Med..

[26] Jansen A (2006). The assessment of hemispheric lateralization in functional MRI–robustness and reproducibility. NeuroImage.

[27] Booth JR (2002). Modality independence of word comprehension. Hum. Brain Mapp..

[28] Chou TL, Chen CW, Wu MY, Booth JR (2009). The role of inferior frontal gyrus and inferior parietal lobule in semantic processing of Chinese characters. Exp. Brain Res..

[29] Chou T-L (2006). Developmental and skill effects on the neural correlates of semantic processing to visually presented words. Hum. Brain Mapp.

[30] Chen PJ (2013). The deficits on a cortical-subcortical loop of meaning processing in schizophrenia. Neuroreport.

[31] Kuperberg GR, Deckersbach T, Holt DJ, Goff D, West WC (2007). Increased temporal and prefrontal activity in response to semantic associations in schizophrenia. Arch. Gen. Psychiatry.

[32] Fan L-Y (2020). Developmental differences of structural connectivity and effective connectivity in semantic judgments of Chinese characters. Front. Hum. Neurosci.

[33] Fan LY, Lee SH, Chou TL (2010). Interaction between brain regions during semantic processing in Chinese adults. Lang. and Linguist..

[34] Lee S-H, Booth JR, Chen S-Y, Chou T-L (2011). Developmental changes in the inferior frontal cortex for selecting semantic representations. Dev. Cogn. Neurosci.

[35] Wu C-H (2014). Reduced structural integrity and functional lateralization of the dorsal language pathway correlate with hallucinations in schizophrenia: a combined diffusion spectrum imaging and functional magnetic resonance imaging study. Psychiatry Res. Neuroimaging.

[36] Tabachnick, B. G. & Fidell, L. S. *Using multivariate statistics* 6th edn. (Pearson, 2013).

[37] Ford JM, Mathalon DH, Whitfield S, Faustman WO, Roth WT (2002). Reduced communication between frontal and temporal lobes during talking in schizophrenia. Biol. Psychiatry.

[38] Jeong B, Wible CG, Hashimoto R, Kubicki M (2009). Functional and anatomical connectivity abnormalities in left inferior frontal gyrus in schizophrenia. Hum. Brain Mapp.

[39] Kubicki M (2011). Stochastic tractography study of inferior frontal gyrus anatomical connectivity in schizophrenia. NeuroImage.

[40] Li X, Branch CA, Nierenberg J, Delisi LE (2010). Disturbed functional connectivity of cortical activation during semantic discrimination in patients with schizophrenia and subjects at genetic high-risk. Brain Imaging behav..

[41] Park IH, Park HJ, Chun JW, Kim EY, Kim JJ (2008). Prefrontal functional dissociation in the semantic network of patients with schizophrenia. Neuroreport.

[42] Kopecek M (2007). 18FDG PET in hallucinating and non-hallucinating patients. Neuro Endocrinol. Lett..

[43] Sommer IEC (2008). Auditory verbal hallucinations predominantly activate the right inferior frontal area. Brain.

[44] Weiss EM (2006). Language lateralization in unmedicated patients during an acute episode of schizophrenia: a functional MRI study. Psychiatry Res..

[45] Tyburski E, Sokołowski A, Chęć M, Pełka-Wysiecka J, Samochowiec A (2015). Neuropsychological characteristics of verbal and non-verbal fluency in schizophrenia patients. Arch. Psychiatr. Nurs..

[46] Phelps EA, Hyder F, Blamire AM, Shulman RG (1997). FMRI of the prefrontal cortex during overt verbal fluency. Neuroreport.

[47] Kapur S, Zipursky R, Jones C, Remington G, Houle S (2000). Relationship between dopamine D(2) occupancy, clinical response, and side effects: a double-blind PET study of first-episode schizophrenia. Am. J. Psychiatry.

[48] Liu C-C (2013). Aripiprazole for drug-naive or antipsychotic-short-exposure subjects with ultra-high risk state and first-episode psychosis: an open-label study. J. Clin. Psychoparmacol..

[49] Hsieh MH (2019). Auditory event-related potentials in antipsychotic-free subjects with ultra-high-risk state and first-episode psychosis. Front. Psychiatry.

[50] American Psychiatric Association. *Diagnostic and Statistical Manual of Mental Disorders*. 4th edn (American Psychiatric Association, 2000).

[51] McGorry PD, Yung AR, Phillips LJ (2003). The “close-in” or ultra high-risk model: a safe and effective strategy for research and clinical intervention in prepsychotic mental disorder. Schizophr. Bull..

[52] Cheng JJ, Ho H, Chang CJ, Lane SY, Hwu HG (1996). Positive and negative syndrome scale (PANSS): establishment and reliability study of a Mandarin Chinese language version. Chin. Psychiatry.

[53] Burock MA, Buckner RL, Woldorff MG, Rosen BR, Dale AM (1998). Randomized event-related experimental designs allow for extremely rapid presentation rates using functional MRI. Neuroreport.

[54] Wilke M, Lidzba K (2007). LI-tool: a new toolbox to assess lateralization in functional MR-data. J. Neurosci. Methods.

[55] Hwu HG (2002). Symptom patterns and subgrouping of schizophrenic patients: significance of negative symptoms assessed on admission. Schizophr. Res..

