# Supplementary material for: Hemispheric lateralization of semantic processing before and after aripiprazole treatment in first-episode psychosis or ultra-high risk state
Source: Schizophrenia (Heidelb). 2022 Dec 3;8(1):108. doi: 10.1038/s41537-022-00304-1 (PMC9719558; doi:10.1038/s41537-022-00304-1)
Supplement: Supplementary file 1 — Hemispheric lateralization of semantic processing before and after aripiprazole treatment in first-episode psychosis or ultra-high risk state [file 41537_2022_304_MOESM1_ESM.docx]

**Supplementary material**

Title: **Hemispheric lateralization of semantic processing before and after aripiprazole treatment in first-episode psychosis or ultra-high risk state**

**Supplementary Methods: Stimulus characteristics**

**Supplementary table and figure**

**Table S1.** Between-interval analysis (first vs. second time fMRI) of brain activation for the related vs. perceptual control condition during the semantic task in normal controls (*n* = 6)

**Figure S1.** Spearman’s rank correlations (*r_s_*) among LI value of IFG, accuracy in the semantic judgment task, PANSS scores, and categorical verbal fluency scores in the patient group.

**Supplementary Methods: Stimulus characteristics**

Lexical variables were controlled across the related and unrelated conditions. First, all characters were monosyllabic. Second, the first and second character did not share radicals. Third, the first and second character together did not form a word^1,2^. Fourth, characters were matched for visual complexity (in terms of the number of strokes per character) across conditions. Fifth, characters were matched for frequency across conditions^2^. Sixth, the number of nouns (48–50%), verbs (23%), and adjectives (21–27%) was matched across conditions^1^. The correlation of character frequency or semantic relation with association strength was not significant, indicating that association effects should not be due to differences in frequency or semantic relation^3^.

**References**

1 Corpus, S. Academia Sinica balanced corpus (version). *Taipei, Taiwan* (1998).

2 Wu, J.-T. & Liu, I.-M. Exploring the phonetic and semantic features of Chinese words. *Taiwan National Science Council: Technical Report NSC75-0301-H002-024* (1987).

3 Lee, S.-H., Chen, S.-Y. & Chou, T.-L. Effect of vocabulary size on semantic processing of Chinese characters for fifth graders and adults. *Formos. J. Ment. Health* **22***,* 345-382 (2009).

**Table S1.** Between-interval whole brain analysis (first vs. second time fMRI) of brain activation during the semantic task (related condition vs. perceptual control) in normal controls (*n* = 6)

| **Cortical Regions** | **H** | **BA** | **Voxels** | ***z* test** | **MNI Coordinates** | | |
| --- | --- | --- | --- | --- | --- | --- | --- |
|  |  |  |  |  | ***x*** | ***y*** | ***z*** |
| **Normal controls** | | | | | | | |
| *first time > second time* | | | | | | | |
| Cingulate Gyrus | R | 31 | 236 | 3.98 | 18 | -22 | 40 |
| Superior Temporal Gyrus | R | 22 | 249 | 3.86 | 63 | -46 | 4 |
| Cingulate Gyrus | L | 23 | 115 | 3.78 | -3 | -67 | 31 |
| Superior Temporal Gyrus | L | 22 | 32 | 3.45 | -33 | -31 | 13 |
| Insula | R | 13 | 461 | 3.44 | 39 | -19 | 4 |
| Superior Frontal Gyrus | R | 8 | 39 | 3.32 | 6 | 38 | 61 |

*Note.* H: hemisphere; L: left; R: right, BA: Brodmann’s area; Coordinates of activation peak(s) within a region based on a *z* test are given in the MNI stereotactic space (x, y, z); Voxels: number of voxels in cluster at *p <* 0.005 (uncorrected) with a cluster size greater than or equal to 10 voxels at a whole-brain analysis. FEP: first-episode psychosis; UHR: ultra-high risk state.


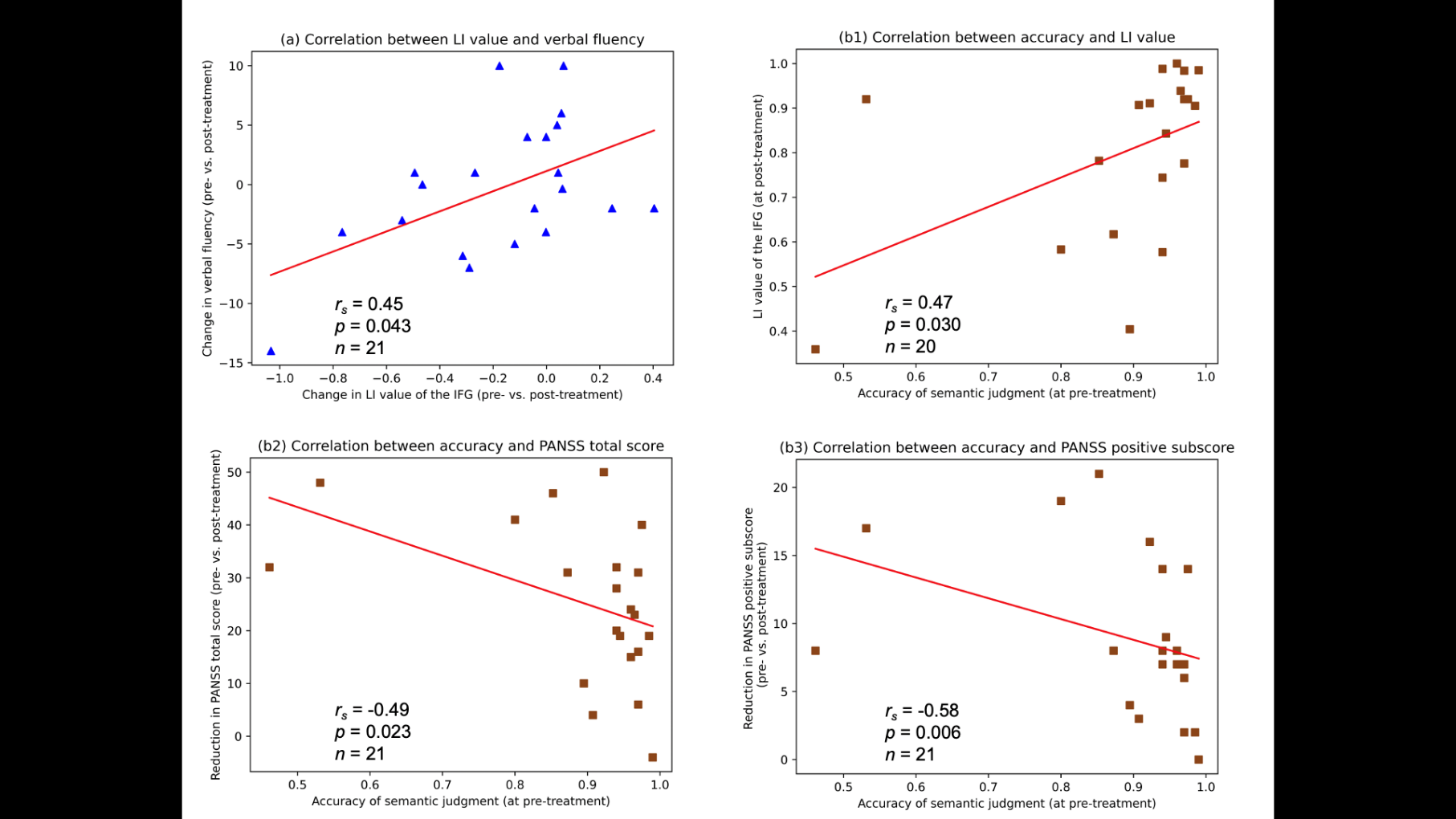

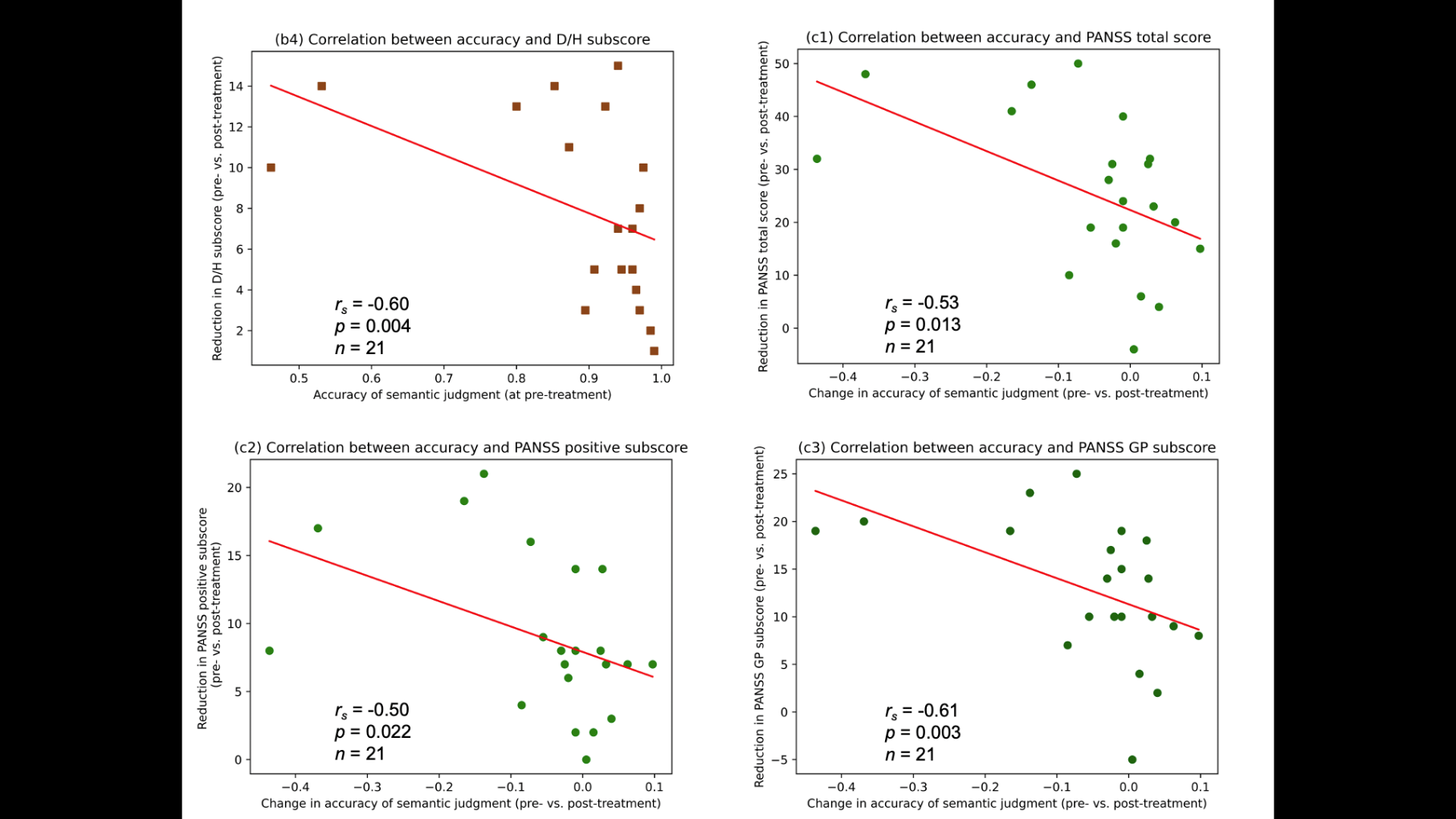


**Figure S1.** Spearman’s rank correlations (*r_s_*) among LI value of IFG, accuracy in the semantic judgment task, PANSS scores, and categorical verbal fluency scores in the patient group: The correlation between change in the categorical verbal fluency score (pre- vs. post-treatment) and change in LI of the IFG (pre- vs. post-treatment)(a); the correlations between accuracy of the semantic judgment task (pre-treatment) and post-treatment LI of the IFG (b1), the reduction in PANSS total score, positive subscore, and hallucination/delusion dimensional subscore (b2-4); the correlations between change in accuracy of the semantic judgment task (pre- vs. post-treatment) and the reduction in the PANSS total score, positive subscore, and general psychopathology subscore (c1-3). It is noteworthy that all scatter plots were based on 21 patients except (b1) in which one outlier was detected based on Mahalanobis distance (corresponding *p* value < 0.001) and Cook’s distance (< 1)^36^. LI: lateralization index; IFG: inferior frontal gyrus; PANSS: Positive and Negative Syndrome Scale.
